# A Novel Sensor-Assisted RFID-Based Indoor Tracking System for the Elderly Living Alone

**DOI:** 10.3390/s111110094

**Published:** 2011-10-25

**Authors:** Chien-Chang Hsu, Jun-Hao Chen

**Affiliations:** Department of Computer Science and Information Engineering, Fu-Jen Catholic University, 510 Chung Cheng Rd., Hsinchuang Dist., New Taipei City 242, Taiwan; E-Mail: jhchen@csie.fju.edu.tw

**Keywords:** elderly living alone, indoor tracking, RFID, three-axis accelerometer, genetic algorithm

## Abstract

The population of elderly people is increasing rapidly in many developed nations. Providing safe and comfortable care to aging people is an important social goal. Moreover, obtaining correct activity and location information for an elderly person is an important research goal. This work proposes a novel intelligent RFID-based indoor tracking system for elderly people living alone. The proposed system uses environment information for inhabitants and received signal strength of an RFID reader to estimate the probable location of an inhabitant. The proposed system then coordinates with the wireless sensor node of a three-axis accelerometer and uses a genetic algorithm to compute the location of the inhabitant. The proposed system also uses context and gait information to improve inhabitant-tracking accuracy. Experiment results show that the accuracy of the proposed system is better than that of existing RFID-based systems.

## 1. Introduction

Advances in medical treatment and health care have increased the population of elderly people in many nations. Many of these elderly people now live alone. However, their degenerating physical functions severely reduce their mobility. Additionally, providing timely care or treatment when indoor accidents occur is difficult. Thus, monitoring the physical location of elderly residents and the surrounding environment to prevent accidents is necessary [1–10]. Many researchers have attempted to achieve this goal by applying intelligent technologies, such as image surveillance, sound detection, or vibration sensing, to identify fall or trip hazards [11–16]. For instance, Zhou *et al.* tracked and identified elderly resident behavior using images [17]. Mukhopadhyay *et al.* created an environment equipped with sensors and robots for elderly care [18]. They monitored physiological signs and remotely monitored the surrounding environment of an elderly person to minimize risk. Using images is the easiest way to track and monitor the movements of elderly people. However, not all are willing to live under surveillance. Monitoring human daily life may invade personal privacy. Thus, an indoor tracking system that considers privacy, safety, and cost is needed.

Other sensors, such as pressure sensors, and radio frequency identification (RFID) are frequently used to detect the locations of inhabitants [19–24]. Various RFID-based localization and tracking methods have been developed [25]. Generally, RFID-based indoor localization methods are categorized as direct, distance, and hybrid measurement methods. Direct measurement directly locates an inhabitant’s position based on the existence or strength of sensor signals. The advantage of this method is that it does not require complex algorithms. However, highly accurate sensors are needed to locate an inhabitant’s position. For instance, Tesoriero *et al.* deployed passive RFID tags on a floor surface and used an RFID reader attached to a robot to detect the grid of tags representing a location unit [26]. Shiraishi *et al.* used a passive ultra-high-frequency (UHF) RFID system to locate the position of inhabitants [27]. Passive UHF tags were deployed in a grid pattern on a ceiling. Users carried RFID readers to receive signals from ceiling tags and to identify their approximate locations. Later, the clustering method and time-average clustering method were used to eliminate false tags and increase accuracy. Alippi *et al.* used several passive RFID readers deployed in an environment to detect the positions of inhabitants via tag signals [28]. Signal strength of passive RFID tags can vary with distance and angles between tags and readers. Using Bayesian theory, Alippi *et al.* located the probable positions and established a probability map for each passive RFID reader. These probability maps were then introduced to the passive RFID readers in an actual environment to identify the most probable location of a user. Jiang *et al.* developed a virtual route-tracking algorithm to track RFID tags and identify a resident’s path [29]. Virtual routes can determine tag paths based on the relationship between neighboring detected readers. Zah *et al.* designed a scout floor with embedded high-frequency and super-high-frequency RFID tags for location detection [30]. Users can carry RFID readers to locate and track the embedded tags under the floor based on the existence of signals. Distance measurement uses signal strength to determine the distance between readers and tags. Detection algorithms are then applied to identify inhabitant positions. For instance, Ni *et al.* developed a LANDMARC (location identification scheme based on dynamic active RFID calibration) system to detect tags in a room [31]. Several active RFID readers, reference tags, and unknown tags were deployed in a room. Euclidian distance was applied to compute differences in signal strength between reference tags and unknown tags. The k-nearest neighbor algorithm was used to search for k-reference tags with the shortest Euclidian distances. The location of the unknown tags was calculated from these reference tags. The major drawback of the LANDMARC scheme is that locations and number of readers and reference tags affect localization accuracy of unknown tags. Some researchers have improved the LANDMARC approach. For example, Jin *et al.* proposed a k effective reference tags algorithm to reduce LANDMARC calculation time [32]. The algorithm computes the Euclidean distance in the same manner as the LANDMARC scheme, and then selects k neighboring reference tags with the smallest weights from the area of reference tags. Finally, the approach uses triangulation to determine the average deviation of every three reference tags to find the final position. Zhao *et al.* proposed a new tracking method called virtual reference elimination [33]. This method uses known reference tags to establish virtual reference tags and eliminate excess readers and tags. Reference tags are deployed along a nine-patch pattern. A proximity map divides the entire sensing area into several regions, where each region represents the area of each virtual reference tag. Based on the Received Signal Strength Indication (RSSI) values of unknown tags, each reader approximates the possible regions of virtual tags and establishes its own proximity map to select the most probable map. Bekkali *et al.* used two active RFID readers and several active RFID tags as reference points to locate user positions [34]. The reference points and user signal strengths received by the two active RFID readers are converted into corresponding distances. The cosine law, triangulation, and a probability distribution function are applied to locate the positions of resident. Shih *et al.* deployed four active readers and several active tags along a grid pattern [35]. Four reference points nearest the tracking tags are spotted, and together form a square within which unknown tags are located. However, most of these LANDMARC-based approaches ignore interference in reader-to-reader, reader-to-tag, and tag-to-tag communication. In addition to using a single sensor type for localization, the hybrid measurement method uses several different sensor types to increase positioning accuracy. Yeh *et al.* installed a pressure sensor, ultrasound receivers, an accelerometer, a three-dimensional orientation sensor, and passive RFID readers on clogs to detect the paths and positions of a user [36]. The combination of these five devices improves localization accuracy. To further minimize detection errors, passive RFID tags are deployed in an environment, such that passive RFID readers can correct signals from tags.

However, most of these methods have problems. First, installing a large number of sensors is costly; the cost to install indoor cameras and pressure sensors is very high [25,37,38]. These devices cannot be applied to ordinary families, especially for the elderly people who live alone. Second, radio signals are adversely affected by interference and are not cost-effective. Additionally, microwave and UHF signals are susceptible to interference from water and metals. Furniture can also reduce location tracking accuracy. Third, attaching an RFID reader to an elderly person is not feasible for location tracking indoors. Finally, sensing range and the electromagnetic waves of RFID readers must be considered. Therefore, an RFID-based location system should have a limited number of sensors to locate positions cheaply and accurately. This work uses a novel sensor-assisted RFID system for indoor environments to locate elderly people living alone.

The proposed system comprises a wireless accelerometer, an active RFID reader with a signal strength function, and some RFID reference tags with environment data to identify the location of elderly people living alone. The proposed system also uses a genetic algorithm to search for the most probable intersection point in data. The wireless accelerometer determines whether a resident is walking. The RFID reader and reference tags estimate a resident’s position and identify walkable paths based on room layout. The system uses a small number of RFID reader and tags to reduce interference and deployment costs, and increases positioning accuracy by integrating contextual information and genetic operators.

The remainder of this paper is organized as follows. Section 2 introduces the proposed sensor-assisted RFID-based inhabitant tracking system. Section 3 discusses system simulation results. Section 4 presents discussions, conclusions, and directions for future work.

## 2. System Architecture

**Figure 1.**
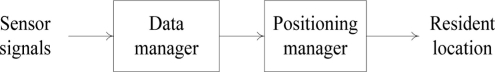
System architecture.

Figure 1 shows the architecture of the proposed sensor-assisted RFID-based indoor tracking system for elderly people living alone. The system has two modules: a data manager and positioning manager. The former filters out noise from sensor data, and determines the probable locations of a resident based on his/her current paths and number of steps. The latter calculates the actual location of residents using location information. The functionality of each module is discussed in the subsections that follow.

### 2.1. Data Manager

The data manager is responsible for signal filtering, walking path estimation, and counting walking steps. The data manager first uses moving average filtering to eliminate noise and smooth received signals. The moving average filter computes the average current sampling value in the sliding window, sums (2*n* + 1) the number of signals before and after current sampling point *i*, and then divides the summed value by number of signals sampled:

(1) $$F_{i}=\frac{\sum_{j=i-n}^{i+n}S_{j}}{2n+1}$$

where F_i_ is the ith moving average value, S is the sampling signals, and *n* is the number of signals sampled in the sliding window.

**Figure 2.**
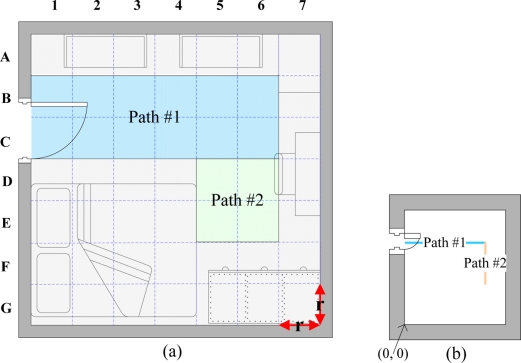
**(a)** Example of paths in a bedroom; and **(b)** minaturized paths in a bedroom.

**Figure 3.**
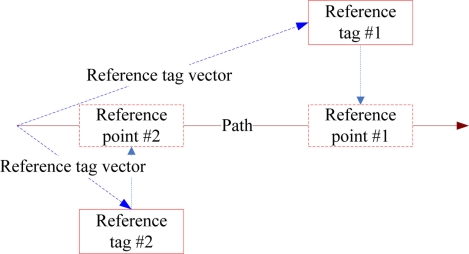
Example of reference tags projected on the path.

**Figure 4.**
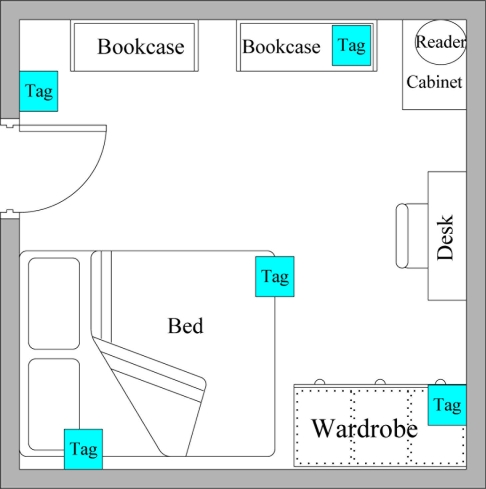
Example of reference tags deployment.

The walking path estimation is based on indoor layout and RFID data. Indoor layout encompasses the indoor activity space and defined occupied objects in the house, such as partitions, sofa, tables, chairs, and wardrobes. The activity space in a room is divided into square grids [Figure 2(a)]. The side length of each square is r units. Walking paths are composed of a number of square patterns. Straight lines are walkable paths [Figure 2(b)]. The size of a path in the figure is minaturized using the scale of 1/2r. Notably, the linear equations for paths 1 and 2 are:

(2) $$\{\begin{array}{l} X=3,a\leq X\leq b\\ Y=3,c\leq Y\leq d \end{array}$$

where (a,b) and (c,d) are the value intervals of the X- and Y-axis, respectively. The RFID data provide signal reference information from various RFID information sources. The vector projection method is applied to obtain the coordinates of reference points on paths (Figure 3). The interpolation method is then used to compute radio signal strength (RSS) of reference point i:

(3) $$R\mathrm{SS}(i)=\frac{\left(D_{i}-D_{0})(S_{i}-S_{0}\right)}{D_{i}-D_{0}}+S_{0}$$

where D_i_, D_0_, and S_0_ are the Euclidian distance between reference point i and the reader, the Euclidian distance between the initial point and the reader, and signal strength of the initial point, respectively. Each room corner has five indoor tags (Figure 4). One tag is placed on each wall and one is placed at the room center. To stop reference points from overlapping, tags are not deployed on the same straight line.

The step movement uses an accelerometer to determine whether a user has taken a step:

(4) $$\mathrm{Step}=\{\begin{array}{l} \text{Yes},C_{1}V_{z}+C_{2}V_{X}V_{Y}-\theta >0\\ \text{No},C_{1}V_{Z}+C_{2}V_{X}V_{Y}-\theta \leq 0 \end{array}$$

where *V_X_*, *V_Y_*, *V_Z_*, *C*, and θ are variation in the X-axis, variation in the Y-axis, variation in the Z-axis, a constant value, and the accelerometer threshold value, respectively.

### 2.2. Positioning Manager

The positioning manager assesses the environment in which a resident lives and locates the positions of residents. The manager calculates the difference in signal strength between reference points and the resident’s tag. The manager then calculates the approximate location (L_XY_) of the resident on minified paths and estimates the resident’s probable zone (Z_XY_) based on the shrinkage ratio of 1/2r:

(5) $$L_{\text{XY}}\times 2r-r\leq Z_{\text{XY}}\leq L_{\text{XY}}\times 2r+r$$

(6) $$L_{\text{XY}}=\sum_{k=1}^{m}\left(W_{k}T_{k}\right)$$

(7) $$W_{i}=\frac{E_{i}}{\sum_{k=1}^{m}E},i\in (1,m)$$

where W, T, and E are the weighted value of signal strength between the resident tag and reference points, the coordinates of reference points, and the difference between signal strength of reference points and the resident’s tag, respectively.

**Figure 5.**
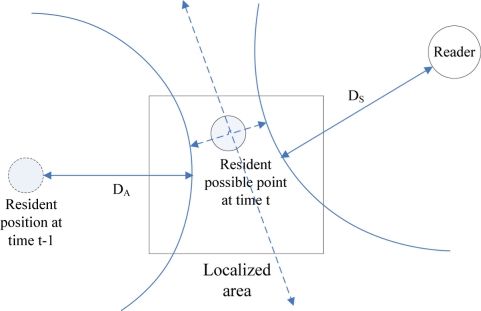
Localization diagram.

Based on information obtained through approximate location, the step size of the resident, and signal strength of the resident’s tag, the genetic algorithm calculates the probable coordinates P_XY_ of the resident (Figure 5). The genetic algorithm encodes these probable coordinates P_XY_ and randomly selects one among these coordinates. The algorithm makes an assessment based on the distance error between the estimated position of resident tag and RFID reader, and the distance difference between resident positions at times (t) and (t − 1):

(8) $$\text{Maximum}\left(\frac{1}{{\left(\Delta D-D_{S}\right)}^{2}+{\left({\Delta D}_{A}-D_{A}\right)}^{2}}\right)$$

where ΔD, D_s_, ΔD_A_, and D_A_ are the Euclidian distance between the resident’s position at time (t − 1) and RFID readers, the distance between the resident and reader according to signal strength of the resident’s tag, the Euclidian distance between the resident’s positions at time (t) and (t − 1), and step size of the resident.

**Figure 6.**
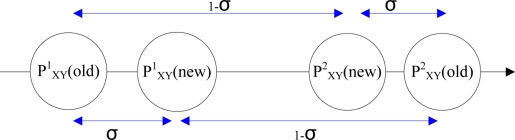
Crossover operation.

When the error of the approximate location exceeds the tolerance threshold, three possible residents’ coordinates with the largest evaluation functions are selected as parent generations for crossover. A minor shift value, *σ*, is added to the three chromosomes with largest evaluation value to generate six child chromosomes as probable coordinates of the resident. Figure 6 shows the cross operation for chromosomes 1 and 2:

(9) $$\{\begin{array}{l} P_{\mathrm{XY}}^{1}(\mathrm{new})=\sigma \times P_{\text{XY}}^{1}(\mathrm{old})+\left(1-\sigma \right)\times P_{\text{XY}}^{2}(\mathrm{old})\\ P_{\text{XY}}^{2}(\mathrm{new})=\sigma \times P_{\text{XY}}^{2}(\mathrm{old})+\left(1-\sigma \right)\times P_{\text{XY}}^{1}(\mathrm{old}) \end{array}$$

where $P_{\mathrm{XY}}^{1}(\mathrm{old})$ is the probable positional coordinate of chromosome 1, $P_{\mathrm{XY}}^{2}(\mathrm{old})$ is the probable positional coordinate of chromosome 2, $P_{\mathrm{XY}}^{1}(\mathrm{new})$ is the probable positional coordinate of chromosome 1 after the crossover operation, and $P_{\mathrm{XY}}^{2}(\mathrm{new})$ is the probable positional coordinate of chromosome 2 after the crossover operation. Notably, the value range of *σ* is (−1, +1) to avoid falling into local optimum. The mutation operation adds noise, *γ*, to the chromosome to shift the probable position to a new position:

(10) $$P_{\mathrm{XY}}^{1}(\mathrm{new})=P_{\mathrm{XY}}^{1}(\mathrm{old})+\gamma$$

where $P_{\mathrm{XY}}^{1}(\mathrm{old})$ is the probable positional coordinate of chromosome 1 and $P_{\mathrm{XY}}^{1}(\mathrm{new})$ is the probable positional coordinate of chromosome 1 after the mutation operation.

**Figure 7.**
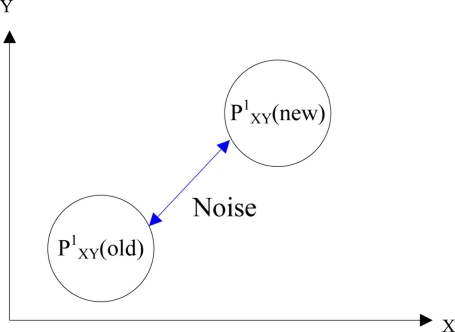
Mutation operation.

Figure 7 shows the mutation operation for chromosome 1. The crossover and mutation probabilities are 0.99 and 0.01, respectively. Finally, when the location search process converges to some positions, computing stops and the location with minimum error is selected as the resident’s location.

## 3. Simulation

**Figure 8.**
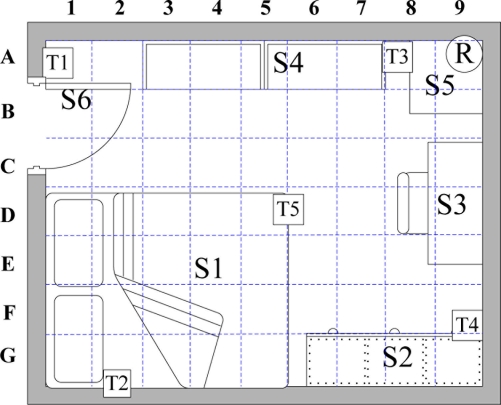
Bedroom layout.

**Figure 9.**
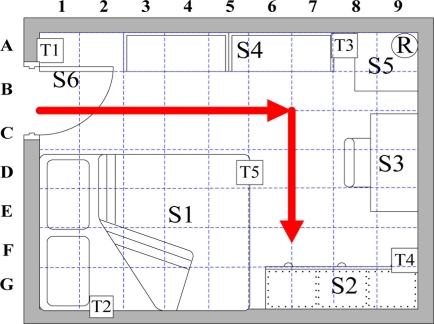
Walking paths of resident.

To track the movement of an elderly person living alone while considering the person’s slow movement, the proposed system monitors an elderly person’s locations in the bedroom, and considers RFID signal interference. Figure 8 and Table 1 show the bedroom layout and the corresponding number of objects in the room of each grid, respectively. The 360 cm × 280 cm bedroom is divided into 63 square grids with the area of 40 cm × 40 cm. Furniture locations are numbered for path establishment. The position of the RFID reader and tags are indicated by a coordinate. The proposed system uses 2.45 GHz active RFID reader and wireless accelerometer with model numbered Freescale ZSTAR3. The development environment is .NET Framework 3.5, C#, and MySQL running on Windows XP. Figure 9 shows the resident’s walking paths. Notably, the starting point is the bedroom entrance, and its coordinate is (0, 200). Resident step length is 40 cm.

### 3.1. Data Manager

**Figure 10.**
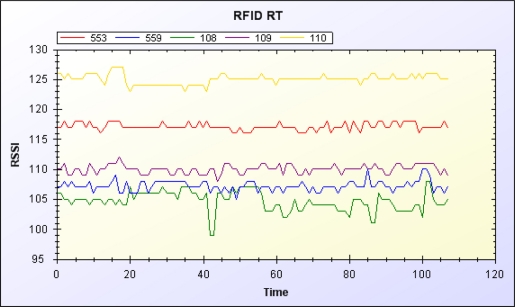
Original RSSI value of RFID.

**Figure 11.**
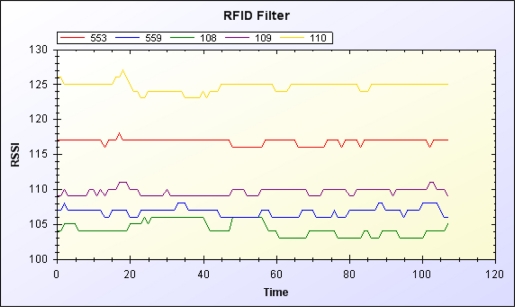
RSSI value of RFID after filtering.

**Figure 12.**
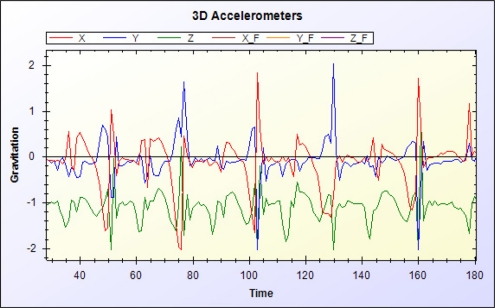
Original acceleration value of tri-axis accelerometer.

**Figure 13.**
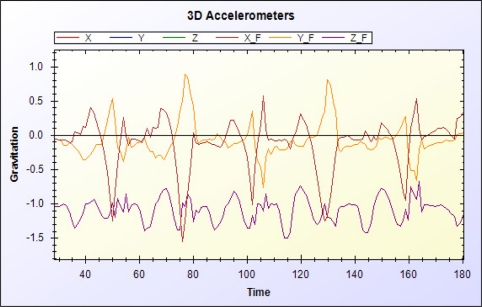
Acceleration value of tri-axis accelerometer after filtering.

The data manager filters signals from RFID readers and the accelerometer. Figures 10 and 11 show the original RSSI value of the RFID and the RSSI value after moving average filtering, respectively. Figures 12 and 13 show the original acceleration value of the accelerometer and the acceleration value after moving average filtering, respectively. When error in the resident’s RFID signal strength is too large, the previous three signal strength variations are used for correction and to overcome signal inaccuracy. Table 2 shows the relationship of RFID signal strength and distance between RFID reader and reference tags.

**Figure 14.**
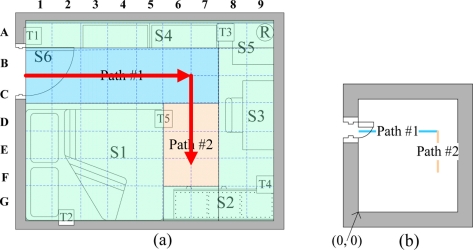
**(a)** Paths in the bedroom; **(b)** Minaturized paths (1/80 scale).

**Figure 15.**
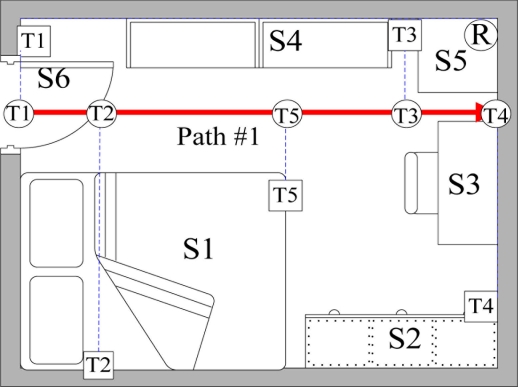
Reference tag projection on path 1.

The data manager establishes walking paths based on the bedroom layout. The furniture layout is in green, while walking paths are blue and orange [Figure 14(a)]. The paths are minified to the scale of 1/80 [Figure 14(b)] and presented with a linear Equation (11). Reference points and the corresponding RSSI values are calculated based on obtained paths (Table 3 and Figure 15).

(11) $$\{\begin{array}{l} y=2.5,\;\;\;0\leq x\leq 3.5\\ x=3.0,\;\;\;0.5\leq y\leq 3 \end{array}$$

The XYZ acceleration variations of the accelerometer after filtering are used to determine number of steps. Acceleration variations (*V_X_*, *V_Y_*, and *V_Z_*), constant value (*C_1_*, *C_2_*) and threshold (*θ*) are (0.6, 0.5, −0.9), (8, −2), and 5, respectively. The step movement is computed in Equation (12):

(12) Step=Yes,8*0.6+(−2)*0.5*(−0.9)−5=0.7>0

### 3.2. Positioning Manager

The first step from the start point is taken as an example. The signal strength of the resident’s tag is 128. Table 4 lists the estimated values of *E_1_*, *E_2_*, *E_3_*, *E_4_*, *E_5_*, *W_1_*, *W_2_*, *W_3_*, *W_4_*, *W_5_*, and *L_XY_*. The probable zone (Z_XY_) of the resident is magnified 80 times in Equation (13):

(13) $$\{\begin{array}{c} 0.44*80-40\leq Z_{x}\leq 0.44*80+40\\ 2.5*80-40\leq Z_{y}\leq 2.5*80+40 \end{array}\Rightarrow \{\begin{array}{l} -4.8\leq Z_{x}\leq 75.2\\ 160\leq Z_{y}\leq 240 \end{array}$$

The real value genetic algorithm is used to calculate the resident’s coordinates. The crossover rate is 0.99, and mutation rate is 0.01. Calculation stops after 1,000 iterations. The following nine sets of coordinates are randomly selected in the first selection: (39.2, 184), (28.2, 201), (34.2, 209), (10.2, 223), (43.2, 184), (−2.8, 236), (47.2, 218), (64.2, 239), and (71.2, 227). The evaluation function is introduced to spot coordinates that match. If no match exists, the first three coordinates with the largest evaluation functions are selected. Table 5 shows the crossover and mutation of resident coordinate encoding. The probable resident coordinate (29, 174) is obtained after 13 crossover and mutation operations. Each circulation contains nine sets of codes; the first four sets with the smallest errors are selected.

### 3.3. Experimental Evaluation

**Figure 16.**
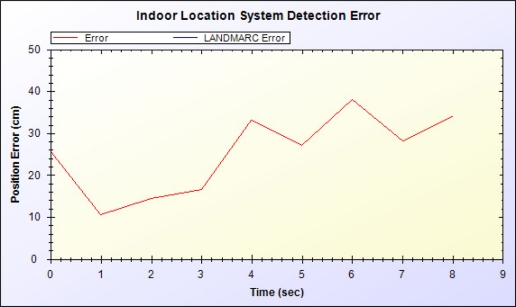
Positioning error diagram.

Figure 16 shows the difference (e) between the estimated location by the proposed system and actual location:

(14) $$e=\sqrt{{\left(x-\text{dx}\right)}^{2}+{\left(y-\text{dy}\right)}^{2}}$$

where (x, y) and (dx, dy) respectively are the calculated XY coordinate and actual XY coordinate. Time 0–4 indicates the five steps on path 1, and time 5–9 indicates the four steps on path 2. As distance from the entrance start point increases, the magnitude of error increases. Each step on path 2 is moved closer to the reader but signal strength of each step is not changed equally. Therefore, determining the approximate location using the ratio of signal strength to RFID reader is inaccurate. Nevertheless, via correction by the accelerometer and genetic algorithm, error and inaccuracy are limited.

## 4. Discussion and Conclusions

### 4.1. Discussion

**Figure 17.**
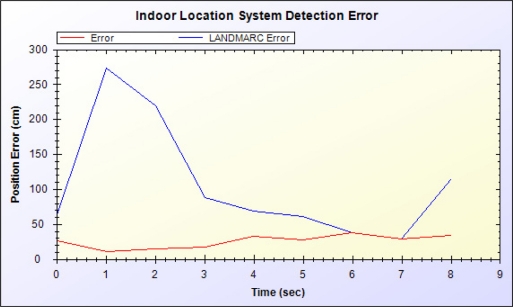
Comparative analysis with LANDMARC.

Figure 17 shows the errors between estimated location and actual location by the proposed sensor-assisted RDIF-based inhabitant tracking system and LANDMARC system. The LANDMARC approach uses only the ratio of signal strength to RFID readers for localization; thus, accuracy is unstable and a large error occurs. The proposed system uses miniaturized paths and an accelerometer to determine whether a resident is moving, thereby markedly increasing accuracy.

Various indoor localization methods exist, some apply only one sensor type and others use multiple sensor types. Direct measurement and distance measurement are the two most common measurement methods. Although direct measurement, which locates positions simply based on sensor signal strength is simple and straightforward, its cost increases as accuracy increases. Additionally, as a sensing area enlarges, the number of sensors required increases, as does cost. Distance measurement locates positions by applying signal strength to determine the error or distance relationship between RFID readers and tags. However, signal strength is susceptible to interference from both the environment and people. No effective solutions currently exist for signal interference. Although distance measurement is much cheaper than direct measurement and requires fewer sensors, the problem of signal interference remains unsolved. This work applies a novel system incorporating distance and smart measurements to correct errors and locate positions. Smart measurement locates positions with only a few sensors. Given that any measurement will inevitably generate errors, smart measurement utilizes smart algorithms for correction and reasoning, thereby improving accuracy significantly.

Table 6 shows previous RFID studies and compares these with the proposed system. Ni used the LANDMARC scheme to locate positions based on the RFID signal strength ratio [31]. Ni determined the ratio of signal strength of unknown tags to that of known tags, and then calculated locations of unknown tags based on those of known tags. The advantage of Ni’s method is that it only requires the RFID signal strength ratio for localization; however, the drawback is inaccuracy caused by too much signal interference. Jin reduced the calculation time of the LANDMARC approach by determining the locations of unknown tags based on overlapping areas of several readers, reducing the time for calculating excess signal strength [32]. Although Jin’s method reduces the calculation time required for the LANDMARC technique, its drawback is inaccuracy caused by too much signal interference. Shih used the signal strength ratio to locate the four known tags nearest unknown tags [35]. The square formed by the four known tags was divided to locate the closest area. The advantage is of this method is that it reduces the number of known tags required in the LANDMARC scheme; however, its drawback is inaccuracy caused by signal interference. Zhao reduced the number of excess known tags in the LANDMARC scheme by applying interpolation to the square formed by the four known tags to establish virtual tags [33]. Proximity maps were generated to determine the probable locations of unknown tags, and the signal strength ratio was then used for positioning. The advantage of this method is that it effectively reduces the number of known tags; however, it still suffers from signal interference. Bekkali utilized signal strength and the corresponding distance between RFID readers and tags to locate positions [24]. Bekkali calculated the angles and distances between readers and tags, and then used the cosine law to calculate the distances between known and unknown tags. Finally, triangulation was applied to obtain the locations of unknown tags and establish a probabilistic RFID map to correct locations. Although Bekkali’s method does not require an excessive number of readers and tags, it requires a large amount of reference tags for location correction.

### 4.2. Conclusions

This work proposes a novel sensor-assisted RFID-based indoor tracking system for elderly people living alone. The proposed system has two modules: the data manager and positioning manager. The data manager filters sensor signals by removing noise. With contextual information and XYZ acceleration values from the accelerometer, the data manager establishes walking paths and determines the number steps taken by a resident. Contextual information includes the layout of the occupied objects in the room, locations and signal strengths of RFID sensors, and XYZ acceleration values measured by the accelerometer while a resident is walking. The positioning manager uses the RSSI value of reference tags and walking paths to calculate approximate regions. Based on a resident’s RFID signal strength, number of steps, and approximate regions, the positioning manager uses the genetic algorithm to determine the probable locations of a resident.

The primary features of the proposed system are as follows. First, the system estimates probable regions of a resident, and then uses the location optimization method to find the locations with the smallest error. The proposed approach uses environment data to establish walking paths and incorporates the signal strength ratio to identify probable resident locations, thereby reducing the possibility of error and reducing the size of the probable area. With the tri-axis accelerometer, the approach corrects the coordinate of the actual location based on the number of steps taken and resident step length. Finally, the proposed scheme uses the real-value genetic algorithm to eliminate uncertain data and locate probable resident positions.

## 5. Future Work

The proposed intelligent RFID-based indoor tracking system only uses two sensor types, and the accelerometer determines whether a resident is walking. Therefore, little usable information for indoor tracking is generated. We suggest that future research integrate other sensors types. Additional resident information will enrich positioning data and improve the accuracy of location tracking. Furthermore, the RFID system acquires data from tags via radio frequencies. These radio frequencies can be adversely affected by interference in indoor environments. The reading rate depends markedly on the experience of an RFID system engineer placing or adjusting RFID readers and reference tags. Developing an intelligent system that automatically deploys readers and tags indoors is also a worthy research goal. Moreover, automatic collection of physiological data from elderly people living alone is another worthy research direction in modern society. Integration of RFID tags with other physiological sensors can extend the amount of time the elderly can live alone in their homes. Applications include health promotion and homecare for the elderly. For instance, the proposed system can prescribe appropriate physical exercises for the elderly according to indoor location, and monitor the indoor activity of the elderly.

## Figures and Tables

**Table 1.** Context information of bedroom.

**Environment layout**	**Number**	**Location**
Bed	S1	D1, D2, D3, D4, D5, E1, E2, E3, E4, E5, F1, F2, F3, F4, F5, G1, G2, G3, G4, G5
Wardrobe	S2	G6, G7, G8, G9
Desk	S3	C8, C9, D8, D9, E8, E9
Bookcase	S4	A3, A4, A5, A6, A7
Cabinet	S5	A8, A9, B8, B9
Door	S6	
Others		A1, A2, F8, F9
RFID reader	R	(360, 280)
Reference tag #^1^	T1	(0, 260)
Reference tag #^2^	T2	(60, 0)
Reference tag #^3^	T3	(280, 280)
Reference tag #^4^	T4	(360, 40)
Reference tag #^5^	T5	(200, 140)

**Table 2.** Relation between RFID signal strength and distance.

**Reference tag #**	**Distance between reader and tag (CM)**	**RSSI**
1	360	127
2	410	117
3	80	161
4	240	136
5	210	140

**Table 3.** Coordinates and signal strength of reference points on path 1.

**Reference tag**	**Coordinates of reference point**	**Minified coordinates of reference point**	**Signal strength of reference point**
T1	(0, 200)	(0, 2.5)	125.24
T2	(60, 200)	(0.75, 2.5)	135.71
T3	(280, 200)	(3.5, 2.5)	155.65
T4	(360, 200)	(4.5, 2.5)	161
T5	(200, 200)	(2.5, 2.5)	145.03

**Table 4.** Estimation of location parameters.

**E1**	**E2**	**E3**	**E4**	**E5**	**L**
0.1312	0.1316	0.0013	0.0009	0.0034	
W1	W2	W3	W4	W5	(0.44, 2.5)
0.4807	0.4986	0.0048	0.0034	0.0013	

**Table 5.** Example encoding process of genetic algorithm.

**Generation #**	**Location 1**	**Location 2**	**Location 3**	**Location 4**
0	(39.2, 184)	(28.2, 201)	(34.2, 209)	(10.2, 223)
1	(37, 188)	(36, 192)	(31, 197)	(39.2, 184)
2	(35, 189)	(35, 191)	(33, 194)	(37, 188)
3	(34, 189)	(33, 190)	(35, 189)	(35, 191)
4	(32, 188)	(33, 188)	(34, 189)	(33, 190)
5	(34, 182)	(35, 183)	(32, 188)	(33, 188)
6	(32, 183)	(34, 181)	(34, 182)	(35, 183)
7	(33, 180)	(32, 183)	(32, 183)	(34, 181)
8	(31, 181)	(33, 180)	(31, 182)	(32, 183)
9	(31, 179)	(30, 180)	(31, 181)	(33, 180)
10	(31, 179)	(29, 178)	(28, 177)	(28, 177)
11	(29, 176)	(29, 176)	(31, 179)	(29, 178)
12	(31, 177)	(31, 177)	(29, 176)	(29, 176)
13	(29, 174)	(29, 174)	(29, 176)	(31, 177)

**Table 6.** Tracking related systems comparison.

**Feature**	**Proposed system**	**LANDMARC**	**Jin**	**Shih**	**Zhao**	**Bekkali**
Reader#	1	Multiple	Multiple	4	Multiple	2
Tag#	Few	Many	Many	Medium	Medium	Medium
Interference problem	Handle	Neglect	Neglect	Neglect	Neglect	Neglect
Auxiliary device	Tri-axis accelerometer	None	None	None	None	None
RSSI reference	Yes	Yes	Yes	Yes	Yes	None
Reference style	Reference points	Reference tags	Reference tags	Reference tags	Reference tags	Reference tags
Context information	Yes	None	None	None	None	None
